# Relative deprivation and forgiveness among Chinese college students: a moderated mediation model of ego depletion and psychological capital

**DOI:** 10.1186/s40359-025-03806-6

**Published:** 2025-12-10

**Authors:** Feifei Li, Han Mao, Siyuan Zhang, Xue Lin

**Affiliations:** School of Business, Shandong Normal University, Jinan, Shandong Province 250014 People’s Republic of China

**Keywords:** Relative deprivation, Forgiveness, Ego depletion, Psychological capital, College students

## Abstract

**Background:**

Psychological education among college students has been receiving considerable attention in China. Forgiveness, as a key positive psychological trait, is widely acknowledged as a pivotal element in reducing the presence of adverse affective states and maladaptive conduct, and is concurrently observed alongside higher psychological well-being. Previous research on forgiveness has primarily centered on individual-level factors, particularly in relation to interpersonal offenses, while relatively few studies have examined broader social factors that may constrain forgiveness. This research sought to investigate the association between relative deprivation, an inhibitor rooted in the broader social environment, and forgiveness, highlighting the mediating role of ego depletion and the moderating role of psychological capital.

**Method:**

This research employed a survey questionnaire including scales measuring relative deprivation, forgiveness, ego depletion, psychological capital and relevant control variables. A total of 823 college students across four grade levels completed on-site surveys in Shandong Province, China.

**Result:**

The survey results confirmed the negative relationship between relative deprivation and forgiveness and highlighted the mediating role of ego depletion in this connection. Additionally, psychological capital played a significant moderating role in the association between relative deprivation and ego depletion. Specifically, relative deprivation was more strongly related to ego depletion among Chinese college students with higher levels of psychological capital compared to those with lower levels. Moreover, psychological capital also moderated the indirect pathway from relative deprivation to forgiveness via ego depletion, highlighting its context-dependent nature in shaping these dynamics.

**Conclusion:**

Relative deprivation was linked to forgiveness via ego depletion as a partial mediator. Psychological capital moderated both the association between relative deprivation and ego depletion and ego depletion’s mediating role in the link between relative deprivation and forgiveness.

## Introduction

Emerging research has increasingly indicated a notable vulnerability of Chinese college students to various mental health challenges. Among the 1,681 college students examined cross-sectionally, 56.8% presented with depressive symptoms [1]. Additionally, an empirical study in Hong Kong revealed that 58% of respondents admitted to engaging in cyberbullying, while 68% reported being victims of cyberbullying [2].These findings underscore the urgent need for targeted strategies aimed at enhancing mental health outcomes among this demographic group. In this context, forgiveness has emerged as a valuable psychological resource, with empirical evidence linking it to reductions in depression and anxiety, as well as enhanced resilience and improved coping strategies in interpersonal conflicts [3, 4]. Considering its established significance for young adults, forgiveness-based approaches may prove effective in ameliorating psychological distress among college students [5].

A common conceptualization of forgiveness is that it refers to a transitional process of individuals to give up negative cognition (e.g., hostility, anger, and vindictive thoughts) and negative behaviors (e.g., verbal abuse) towards the offender after being unjustly offended, replacing these with positive ones [6]. With scholars diving into the influences on forgiveness, multiple factors have been identified, such as personality traits [7], psychological adjustment process [8], offense-specific influences [9], relationship-specific influences [10], and individuals’ cultural backgrounds [11]. However, scholars have focused primarily on the factors promoting forgiveness, while ignoring the factors that inhibit forgiveness. As of yet, few studies have systematically examined the outcomes of heightened rumination and adverse emotional states [12], neuroticism [13], asymmetry in the intent of the victim and transgressor [14], thereby limiting the potential for identifying ways to support higher levels of individual forgiveness.

Another constraint lies in the tendency of previous studies to examine forgiveness determinants primarily through interpersonal lenses, rather than delving into how perceptions of the broader macro-social context shape forgiveness. As China is undergoing a stage of social and economic transformation, the widening income gap causes a portion of members psychologically vulnerable to feelings of unfairness and being deprived [15]. Relative deprivation means the perception that individual is disadvantaged when compared with another relevant referent or groups in the larger social context and the feelings of anger and resentment related to the comparison [16]. Recent years have witnessed an ever-increasing focus on the studies of relative deprivation, particularly on how it interacts with Chinese college students and explains their deviant behaviors involving the moral disengagement and aggression [17, 18]. Research of this kind has illustrated not only the detrimental consequences associated with relative deprivation but also its widespread prevalence among college students.

However, it remains unclear whether the prevailing relative deprivation, as a macro-level factor, has a negative bearing on forgiveness, and if so, whether this association varies across individuals. These questions remain inadequately addressed in prior research. To bridge this existing gap in research, we examined the underlying mechanisms linking relative deprivation to forgiveness, aiming to enrich existing literature on college students’ mental health and provide insights for supporting the development of positive psychological traits during this pivotal stage of their lives.

### Relative deprivation and forgiveness

The concept of relative deprivation can be traced back to the seminal work of Stouffer et al. in “The American Soldier”, which revealed that individual satisfaction depends more heavily on social comparison processes than on absolute circumstances [19]. Building upon this foundational insight, Runciman provided systematic conceptualization of relative deprivation, defining it as the perceived gap between what individuals believe they deserve and what they actually receive when comparing themselves with others [20]. Subsequently, Walker and Smith offered a comprehensive theoretical synthesis that consolidated decades of both theoretical development and empirical investigation in this domain, establishing an integrative framework for understanding relative deprivation phenomena [21]. Relative deprivation which is shaped by the judgments of believing oneself to be comparatively disadvantaged and consequently the feelings of resentful and anger is an outcome of upward social comparison [22, 23].College students from diverse economic and social backgrounds across the country are frequently exposed to upward social comparisons in communal living environments [24]. In line with Social Comparison Theory, the tendency to partake in these types of comparisons diminishes as individuals grow older, peaking in young adulthood and gradually declining thereafter [25]. Consequently, college students are especially prone to experiencing relative deprivation.

The impact of relative deprivation on forgiveness can be understood from the following key perspectives. Relative Deprivation Theory indicates that the deprivation of basic rights perceived by disadvantaged individuals during social comparisons primarily generates a sense of unfairness, which can give rise to problems related to psychological adaptation, including feelings of loneliness, depressive symptoms, and social anxiety [16, 26]. This perceived unfairness hinders the likelihood of forgiveness. The stress-and-coping model of interpersonal forgiveness [27] shows that the magnitude of the injustice gap is proportional to the difficulty of forgiving an offense. Studies have consistently shown that as the injustice gap increases, individuals are less inclined to forgive [28, 29]. Research by Strelan, Di Fiore and Prooijen [30] substantiated the link between perceptions of justice and the capacity to forgive. Forgiveness, characterized by reduced retaliation, conflict avoidance, and increased benevolence towards the offender, is less likely to occur in the absence of justice. Moreover, relative deprivation, by fostering a sense of injustice in social comparisons, may fuel motivations for revenge or avoidance of others [27]. Thus, relative deprivation is relevant to the formation of a psychological climate that corresponds to lower the cognitive processes necessary for forgiveness.

From an emotional perspective, such deprivation frequently engenders adverse emotional responses, including resentment and anger, among disadvantaged individuals or groups [16]. Systematic meta-analytic evidence indicates that anger, as the primary negative emotion triggered by deprivation, directly impedes the process of forgiveness [31]. Additionally, relative deprivation is typically marked by an absence of self-blame, where disadvantaged individuals do not attribute their experience of deprivation to themselves or hold themselves accountable for their difficulties [16]. With the lack of self-blame, individuals experiencing relative deprivation may find it difficult to achieve reductions in their negative emotions through positive reframing and are more likely to express their grievances outwardly. Over time, these unresolved emotions tend to accumulate, further restricting their ability to forgive.

Additionally, emerging theoretical perspectives suggest that sustaining harmonious interpersonal relationships represents a core motivational factor in forgiveness processes [32, 33]. The dynamic forgiveness process framework proposed by Ho & Fung [34] highlights the significant role of sociocultural factors in shaping forgiveness. In many collectivist cultures, harmony serves as a major impetus for forgiving behavior [35]. In the Chinese context, other-oriented personality traits, such as face concern, harmony-seeking, and relational orientation, have been shown to correlate more strongly with forgiveness among college students than individualistic personality traits like as self-esteem [7]. However, the perception of relative deprivation generates psychological conflict among college students, as the sense of being deprived compared to others, weakens their collectivist values and diminishes their motivation to uphold interpersonal harmony, ultimately resulting in social alienation [36–38]. Under these conditions, the likelihood of achieving forgiveness through friendly peer relationships becomes relatively low. Taken together, these results indicate that relative deprivation may be in connection with challenges for forgiveness processes.

### Ego depletion as a mediator

People may restrain their natural impulse toward the negative cognitions and emotions in order to forgive others after experiencing deprivation. However, this typically necessitates higher-order cognitive mechanisms, notably including self-regulatory functions [39]. The strength model of self-regulation reveals that self-regulation is a universal yet finite resource individuals employ to consciously exert control over the self [40]. Growing evidence indicates that individuals’ capability for self-regulation is limited, and tasks requiring active volitional control gradually deplete an individual’s self-regulatory resources [41]. This depletion in self-regulatory resources is termed “ego depletion” [40]. In the context of relative deprivation and forgiveness, we infer that ego depletion may act as a mediating variable.

When individuals attempt to manage the negative cognitions and emotions stemming from relative deprivation, they may experience ego depletion. The strength model of self-regulation posits that inhibition of impulses, including conscious override of unwanted thoughts, negative emotions, and behaviors, relies on the same limited self-regulatory resources, which ultimately leads to ego depletion [42]. In the context of relative deprivation, college students’ efforts to suppress feelings of unfairness and negative perceptions of their peers may exhaust these regulatory resources, leading to a state of ego depletion. Empirical evidence for this comes from a study by Osborne, Sibley, and Sengupta [43] which found that college students from lower socioeconomic backgrounds were more likely to experience relative deprivation, resulting in diminished self-regulatory resources. Similarly, Inzlicht and Kang [44] noted that interpersonal stressors, such as perceived deprivation relative to peers, contribute to volitional depletion. Moreover, from an emotional standpoint, emerging studies indicate that elevated perceptions of relative deprivation correlate significantly with heightened anxiety, hostility, and depressive symptoms [45]. Further researches have uncovered a notable link between relative deprivation and envy among Chinese adults [17], in which individuals from disadvantaged circumstances are more likely to harbor malicious envy toward those in more privileged positions. Considering the finite nature of self-regulatory resources, managing these negative emotions may demand substantial effort, which may be a element in the depletion of an individual’s volitional regulatory resources.

When experiencing ego depletion, individuals may encounter greater difficulty in forgiving others. Drawing from the strength-based model of self-regulation, self-regulatory efforts not only give rise to ego depletion but also trigger the negative effects that follow depletion [40, 46]. Ego depletion affects how individuals respond to tasks requiring active volition. A range of studies has shown that people experiencing self-regulatory depletion face greater difficulties controlling maladaptive behaviors, such as aggression [47], prejudice [48], and surface acting [49], compared to those who are not depleted. Therefore, it can be inferred that in situations where individuals feel deprived, prosocial initiatives demanding conscious intent are compromised under conditions of insufficient regulatory resources. For example, employees with depleted self-regulatory resources showed a reduced propensity for participating in citizenship acts within organizations [50]. Similarly, a study by Jin, Kim, Suh, Sheehan, and Meeds [51] revealed that when individuals were in a deprived state, messages focused on self-benefit were more useful than those centered on other-benefit in eliciting supportive behaviors. These findings demonstrate that ego depletion weakens individuals’ ability to suppress egotistic desires and lowers their inclination to prioritize the well-being of others. In turn, forgiveness, which is an inherently prosocial behavior that requires overcoming instinctive feelings of resentment and showing benevolence toward offenders, becomes unlikely to occur in a state of depletion. Furthermore, states of ego depletion prompt individuals to increase their reliance on recognition heuristics, resulting in simpler decision-making strategies [52]. This aligns with adaptive decision-making theory, which posits that individuals are inclined to choose simpler strategies when operating under limited resources [53]. Accordingly, depleted individuals are more inclined to rely on effort-reducing heuristics and may find it increasingly difficult to restrain their impulses and follow prosocial desires, such as forgiveness.

Based on these observations, it may be concluded that people in a deprived state are more inclined to follow instinctual behaviors and are less inclined to engage in effortful actions requiring substantial self-regulation, such as forgiveness.

### Psychological capital as a moderator

Although ego depletion provides an explanation for how relative deprivation influences forgiveness, this effect is likely moderated by individual differences. Psychological capital (PsyCap) is one of the critical resources identified by Lazarus and Folkman [54] as essential for individuals to cope with stressful events, thereby reducing stress-related symptoms. This construct is defined as the constructive psychological developmental state within individuals and is treated as state-like, developable [55]. As a meta-construct, psychological capital encapsulates the shared characteristics across four constituent factors, including hope, resilience, optimism and efficacy. Conceptual and empirical evidence has demonstrated that psychological capital provides added impacts over human and social capital [56].

Both the limited strength model and Conservation of Resources (COR) theory furnish theoretical grounding for the function of psychological capital in this study. In addition to providing theoretical insights into self-regulatory depletion, the limited strength model also highlights that self-regulatory resources can be refilled and stored [57]. A meta-analysis of 83 studies confirmed that motivational incentives and self-control training significantly enhance self-regulatory capacity in depleted individuals [58]. COR theory not simply examines how people cope with resource loss and depletion, but also emphasizes that those with greater resource reserves are less vulnerable to the negative impacts of resource depletion during stressful situations [59]. This suggests that individuals possessing elevated psychological capital can better buffer against the adverse impacts of resource loss, offering resilience in the face of stress and depletion. In view of those theories, if individuals possess new resources to mitigate and compensate for the depletion, negative consequences can be avoided. There is a wealth of evidence suggesting that personal resources can be invested to aid in the process of stress resistance, including self-esteem and self-efficacy [60] and resilience [61]. In the context of ego depletion arising from relative deprivation, given the synergistic effects of psychological capital, individuals can take advantage of it to potentially counteract such depletion, which may be viewed as a buffer against ego depletion.

It has been widely proved that psychological capital can neutralize the negative effects of stressful perceptions. Specifically, this psychological resource proves instrumental in mitigating the adverse impacts of workplace stressors and uncivil behaviors [62]. Evidence is provided by burnout research, which is anchored on the Job Demands-Resources (JD-R) Model. Job burnout, often resulting from energy depletion due to excessive work demands, can be alleviated by intrinsic motivational factors such as resilience and positive affect [63]. In a study conducted with a Chinese sample, researchers highlighted how psychological capital moderated the association between emotional labor and job burnout [64]. Similarly, Kuvaas, Buch, Weibel, Dysvik, and Nerstad (2017) [65] reinforced the significance of intrinsic motives in alleviating the consequences of job burnout. Moreover, job insecurity was proven to negatively affect workers’ subjective well-being and occupational functioning, but individuals who possess elevated psychological capital are more equipped to manage job insecurity [66]. These studies collectively suggest that those endowed with substantial psychological capital possess stronger psychological resources that shield them from the detrimental impacts of stress and emotional labor, functioning as a resilience buffer against burnout.

Based on these detailed discussions, we propose that psychological capital may weaken the link between relative deprivation and ego depletion, and thus adopt it as a moderating variable for examination.

### The present research

**Fig. 1:**
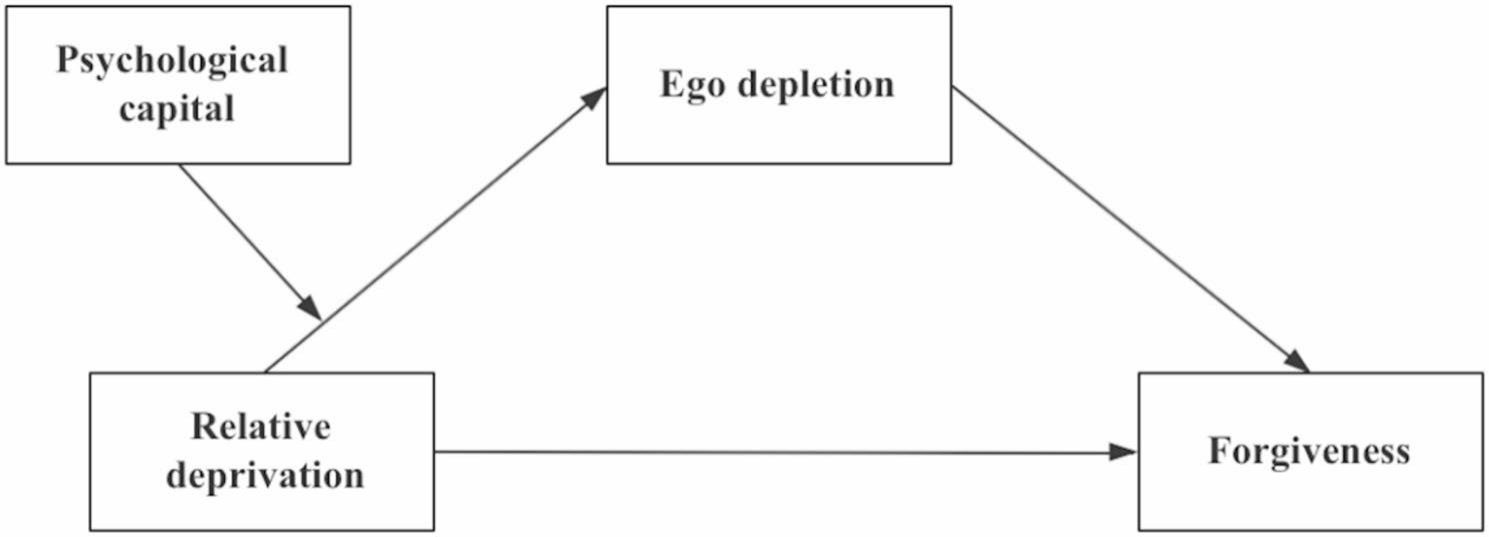
Framework of the moderated mediation model

Drawing from established theoretical perspectives and validated research, this research investigates the association between relative deprivation and forgiveness and proposes a moderated mediation model (Fig. 1). The subsequent hypotheses are proposed:

- H1: Relative deprivation is negatively correlated with forgiveness.
- H2: Ego depletion mediates the relationship between relative deprivation and forgiveness, such that (a) higher levels of relative deprivation predict greater ego depletion and (b) greater ego depletion predicts reduced forgiveness capacity.
- H3: Psychological capital moderates the relationship between relative deprivation and ego depletion, such that college students with higher psychological capital levels are less susceptible to the negative effects of relative deprivation on ego depletion.
- H4: Psychological capital moderates the indirect effect of ego depletion on the relationship between relative deprivation and forgiveness.

## Methods

### Participants and procedures

A sum of 1,000 university students from five universities in Shandong Province, China were recruited to fill the questionnaires anonymously, after obtaining informed consent from both the university administrations and the students. Preceding the collection of data, all participants gave informed consent after being advised of their voluntary participation rights. We conducted the survey on-site at each university using standardized instructional language from September to October in 2023. Specifically, we ensured the anonymity of our participants and lessened their apprehension by emphasizing the absence of correct or incorrect responses.

Of the 1,000 questionnaires distributed, 896 were completed and returned. Data quality screening procedures identified 73 cases with indicators of insufficient effort responses including long string patterns and atypical response time [67], which were excluded from analyses. The final analytic sample included 823 valid responses (valid response rate = 82.3%). The average age of participants was 19.6 years (SD = 1.31). Students in four separate grades (from the first to the fourth) represented 27.6%, 28.7%, 22.2% and 21.5% respectively. With respect to gender distribution, the sample comprised 45.0% male and 55.0% female participants. On the basis of a section in the questionnaire concerning the economic status of their families, 82.6%, 9.6% and 7.8% reported coming from middle-income, low-income and high-income families respectively.

### Measures

Based on the state-trait continuum theory [55], whole trait theory [68], and the cognitive-affective personality system model [69], this study positioned all variables at the level of relatively stable individual characteristics. This methodological choice reflects contemporary psychology’s understanding of psychological characteristic continuity: (1) relative deprivation and psychological capital are established as relatively stable characteristics [16, 55, 70]; (2) although ego depletion is traditionally viewed as a state variable, recent studies indicate it displays stable individual difference characteristics under certain conditions [58, 71, 72]; (3) forgiveness was measured as a trait, assessing individuals’ stable tendencies across situations and time. This positioning supported theoretical and measurement consistency across all variables.

#### Relative deprivation

Relative deprivation was evaluated by the Chinese version of the Personal Relative Deprivation (PRD) scale which consists of four items [73]. Sample items include “Compared to the effort and dedication I put in, my life should have been better than it is now”; “I am at a disadvantage in multiple areas of life compared with people around me”. A six-point Likert scale was employed to measure responses to these items (1 = strongly disagree, 6 = strongly agree). All items were averaged to create a composite score ranging from 1 to 6, with higher mean scores indicating greater levels of relative deprivation. Cronbach’s Alpha was 0.89, and CFA results showed good validity of the scale: *χ*^*2*^*/df* = 3.19, *χ² =6.38*,* df* = 2, NFI = 0.99, CFI = 0.99, IFI = 0.99, TLI = 0.99, RMSEA = 0.05.

#### Ego depletion

We assessed ego depletion utilizing an ego depletion scale developed by Lin and Johnson [74]. This self-report scale comprises five items. Notably, certain items in this scale, such as “I feel drained” and “My willpower is gone,” may reflect relatively persistent states of resource depletion. To verify this assessment, we conducted a supplementary survey among 489 students across the five universities in our research sample. When asked face-to-face whether these items reflected “general daily state or momentary state at a specific time,” 83.4% reported that these items captured their everyday state characteristics rather than momentary states. This supplementary verification supports the appropriateness of using this scale to measure relatively stable individual characteristics. Each item was rated on a 5-point scale (1 = strongly disagree to 5 = strongly agree). An average score was calculated across the five items, with higher mean values indicating a greater degree of ego depletion. Cronbach’s Alpha was 0.88, and the CFA results demonstrated good validity of the scale: *χ*^*2*^*/df* = 2.66, *χ² =7.97*,* df = 3*, NFI = 0.99, CFI = 0.99, IFI = 0.99, TLI = 0.99, RMSEA = 0.05.

#### Psychological capital

The Psychological Capital Questionnaire (PCQ) constructed by Luthans et al. [55] has been widely used in organizational contexts and is primarily applicable for measuring employees’ psychological capital within occupational settings. To more effectively assess the psychological capital of university students, we used the Chinese version of the Psychological Capital Questionnaire developed by Kuo, Sai, and Yinghong [75]. The reliability and validity of this scale have been extensively demonstrated within samples of Chinese college students. It contains four dimensions of optimism, hope, self-efficacy, resilience, with a total of 26 items measured using a seven-point Likert scale (e.g. “I can recover quickly when I encounter setbacks”; “I rarely care about the unpleasant things in life”; “I always see the good side of things”; “I pursue my goals with confidence”). A mean score was computed across all items, with higher values denoting greater psychological capital. Cronbach’s Alpha was 0.93, and the CFA index showed acceptable validity of the scale: *χ*^*2*^*/df* = 3.12, *χ² =830.17*,* df* = 266, NFI = 0.93, CFI = 0.95, IFI = 0.95, TLI = 0.94, RMSEA = 0.05.

#### Forgiveness

Forgiveness was assessed using a scale developed by Yang, Li, and Zheng [76]. This questionnaire, specifically adapted for Chinese college students, is based on the Tendency to Forgive Scale (TTF) and the Heartland Forgiveness Scale (HFS). It comprises eight items spanning two dimensions: forgiveness and revenge. Sample items include “I often quickly forget how others hurt me “and “I seek retribution against those who have harmed me”. Subjects were collected using a seven-point Likert-type scale. Item responses were averaged, with elevated scores indicating stronger forgiveness. Cronbach’s Alpha was 0.81, and the CFA results demonstrated good validity of the scale: *χ*^*2*^*/df* = 2.30, *χ² =29.98*,* df* = 13, NFI = 0.98, CFI = 0.99, IFI = 0.99, TLI = 0.98, RMSEA = 0.04.

#### Covariates

Prior researches have demonstrated the relationship of age and family income to forgiveness, while gender was not consistently discovered to be associated with forgiveness [77]. However, given that gender may have a bearing on ego depletion [78], we included gender, along with grade and family economic status, as control variables in the next analysis to account for any potential variations.

### Statistical analyses

We conducted descriptive statistics, correlation analysis, and common method bias assessment using SPSS 26.0. We employed structural equation modeling (SEM) to test our hypotheses with Amos 24.0. We first performed confirmatory factor analysis (CFA) to validate the measurement model, then examined the structural model with hypothesized relationships [79]. For psychological capital, we created four parceled indicators representing its theoretical dimensions to lower model complexity while preserving its multidimensional structure [80]. All independent and moderating variables were mean-centered to tackle multicollinearity concerns [81]. Mediation and moderated-mediation effects were evaluated using bootstrap procedures in AMOS 24.0. Based on 5,000 bootstrap samples, 95% bias-corrected confidence intervals were calculated to assess the significance of indirect effects. Path significance was judged through examining whether confidence intervals contained zero.

## Results

### Common method bias and measurement model validity

To address common method bias, we implemented procedural and statistical safeguards following recommended procedures [82]. Procedurally, the survey’s anonymous design, standardized instructions across universities, and assurance of no correct answers helped minimize social desirability and evaluation apprehension. We also incorporated reverse-coded items and excluded 73 insufficient-effort responses based on response patterns and completion times. Statistically, we conducted a full collinearity assessment using variance inflation factors (VIFs), a rigorous diagnostic approach [83]. All VIF values fell substantially below the recommended 3.3 threshold: relative deprivation (1.45), ego depletion (1.73), forgiveness (1.14), and psychological capital (1.40). While Harman’s single-factor test yielded 30.3%, we acknowledged the limitations of Harman’s approach [82] and relied primarily on VIF diagnostics as a more rigorous assessment. The combination of procedural controls and VIF results suggests that common method bias is unlikely to substantially distort our findings.

The measurement model’s fit indices were evaluated based on the criteria proposed by previous researchers, which include “chi-square/degree of freedom (CMIN/DF) ≤ 5 [84], CFI ≥ 0.90 [85], RMSEA ≤ 0.08 [86], GFI ≥ 0.90 [87], and IFI ≥ 0.90 [85]. Across fit metrics, the measurement model fell within acceptable bounds: *χ²/df* = 4.94, CFI = 0.93, GFI = 0.91, IFI = 0.93, TLI = 0.91, RMSEA = 0.07.

### Correlation and descriptive statistics

Table 1 presents means, standard deviations, and Pearson correlations in this research. Relative deprivation was positively correlated with ego depletion (*r* = 0.55, *p <* 0.01) but negatively correlated with forgiveness (*r* = -0.21, *p <* 0.01). We also found that ego depletion had a negative association with forgiveness (*r* = -0.23, *p <* 0.01) and psychological capital (*r* = -0.48, *p <* 0.01). Moreover, forgiveness was positively correlated with psychological capital (*r* = 0.33, *p <* 0.01).

**Table 1:** Descriptive statistics and correlations among the variables

	M	SD	1	2	3	4	5	6	7
1.Gender	1.55	0.50	1.00						
2. Grade	2.38	1.10	0.01	1.00					
3. FES	1.98	0.42	-0.04	-0.08*	1.00				
4.Relative deprivation	3.09	1.04	-0.12**	-0.02	-0.09**	1.00			
5. Ego depletion	2.86	0.80	-0.04	-0.03	-0.10**	0.55**	1.00		
6. Forgiveness	3.97	0.87	-0.03	-0.07*	0.10**	-0.21**	-0.23**	1.00	
7. PsyCap	4.70	0.74	-0.00	-0.04	0.15**	-0.28**	-0.48**	0.33**	1.00

*N* = 823. Gender was dummy coded such that 1 = male and 2 = female. Grade (freshman year, sophomore year, junior year, senior year)

*FES* Family Economic Status (poor, average, rich), *PsyCap* Psychological Capital

*p < 0.05

** *p <* 0.01

### Hypotheses testing

#### Mediation analysis

The structural model demonstrated adequate fit to the data, implying that accepted thresholds were met: *χ²/df* = 2.64, CFI = 0.96, GFI = 0.95, TLI = 0.96, IFI = 0.96, RMSEA = 0.05. Age, gender, and family economic status were incorporated as control variables with direct paths to forgiveness. Support for Hypothesis 1 was established through a significant negative direct association between relative deprivation and forgiveness (*β* = -0.303, *p <* 0.001). Specifically, higher relative deprivation levels corresponded to a lower propensity for forgiveness.

Hypothesis 2, which posited that ego depletion accounts for the relationship between relative deprivation and forgiveness, likewise received empirical support. The bootstrapping analysis (*N* = 5000 resamples) results are presented in Table 2. The analysis revealed a significant indirect association (*β* = -0.129, *95% CI* [-0.193, -0.072]). Decomposition of this indirect pathway shows that relative deprivation was significantly associated with higher levels of ego depletion (*β* = 0.602, *p <* 0.001), which was negatively connected with lower levels of forgiveness (*β* = -0.214, *p <* 0.001). The direct association between relative deprivation and forgiveness persisted after accounting for ego depletion (*β* = -0.303, *95% CI* [-0.395, -0.208]), suggesting that ego depletion partially accounts for this relationship. The variance accounted for (VAF) by the indirect path was 29.93%, highlighting the substantial role of ego depletion in the association between relative deprivation and forgiveness.

**Table 2:** Bootstrapping analysis of mediation effects

Effect Type	Path	β	SE	Z	95% Bias-Corrected CI	
					Lower	Upper
Direct Effect​	RD → FORG	-0.303***	0.048	-6.31	-0.395	-0.208
Indirect Effect​​	RD → ED → FORG	-0.129***	0.031	-4.16	-0.193	-0.072
Total Effect​​	RD → FORG	-0.431***	0.039	-11.05	-0.505	-0.351

*RD* Relative Deprivation, *ED* Ego Depletion, *FORG* Forgiveness, *CI* Confidence Interval

*β* = standardized path coefficient

***p < 0.001

#### Moderation analysis

To examine whether psychological capital moderates the relationship between relative deprivation and ego depletion, after controlling for gender, grade, and family economic status, we assessed the interaction relationship by means of structural equation modeling. As shown in Table 3, both relative deprivation *(β* = 0.484, *p <* 0.001) and psychological capital (*β* = -0.321, *p <* 0.001) exhibited significant associations with ego depletion. The interaction term was significant (*β =* 0.097, *p =* 0.004).

**Table 3:** Moderation analysis results

Path	β	SE	Z	*p*
RD → ED​	0.484	0.020	10.621	< 0.001
PsyCap → ED​​	-0.321	0.024	-8.248	< 0.001
RD × PsyCap → ED​	0.097	0.017	2.905	0.004

*RD* Relative Deprivation, *PsyCap* Psychological Capital, *ED* Ego Depletion

*β =* standardized path coefficient

**Fig. 2:**
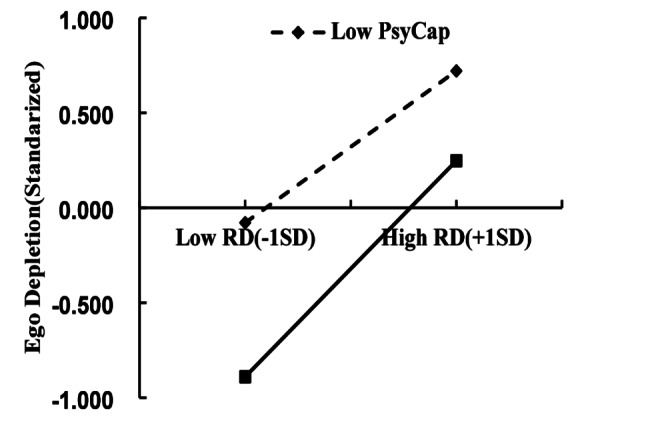
The moderating role of psychological capital. Note: RD = Relative Deprivation, PsyCap = Psychological Capital

Simple slope analysis was conducted to interpret this interaction. The association between relative deprivation and ego depletion was stronger for individuals with high psychological capital (*β =* 0.569, *95% CI* [0.484, 0.648], *p =* 0.001) compared to those with low psychological capital *(β =* 0.400, *95% CI* [0.268, 0.515], *p* < 0.001). Figure 2 presented this pattern, whereby psychological capital strengthened rather than weakened the link between relative deprivation and ego depletion. Unexpectedly, the moderation relationship proved positive instead of the predicted negative, contradicting H3.

#### Moderated mediation analysis

**Fig. 3:**
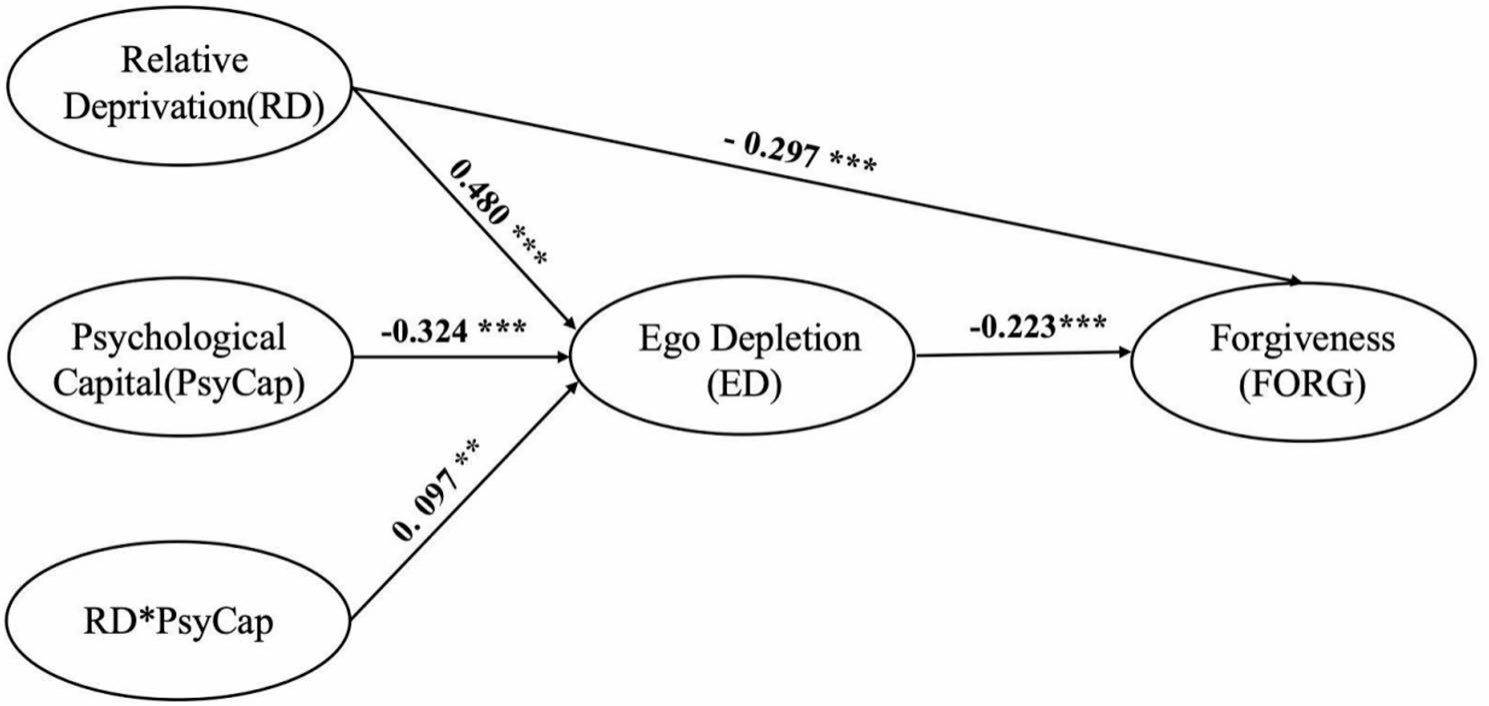
The moderated mediation model. Note: *N* = 823. *** *p* < 0.001, ** *p* < 0.01

Building on the moderation findings, we tested whether psychological capital also moderates the association between relative deprivation and forgiveness through its link with ego depletion after controlling for gender, grade, and family economic status. The integrated moderated mediation model demonstrated acceptable fit to the data (*χ²/df* = 3.83, CFI = 0.91, GFI = 0.90, IFI = 0.92, TLI = 0.90, RMSEA = 0.06). Bootstrap analysis (*N* = 5,000 resamples) showed that all paths in the moderated mediation model reached statistical significance (see Fig. 3). The interaction term remained significant in this expanded model (*β* = 0.097, *p* = 0.004).

The conditional indirect effects analysis confirmed moderated mediation(see Table 4). For individuals with high psychological capital, the indirect effect was *β =* -0.128, *95% CI* [-0.193, -0.073], *p* < 0.001. For those with low psychological capital levels, this indirect effect was *β =* -0.085, *95% CI* [-0.143, -0.045], *p* < 0.001. Neither confidence interval encompassed zero, indicating significant indirect associations at both levels of the moderator. Moreover, the index of moderated mediation was significant (Index = -0.043, *95% CI* [-0.092, -0.013], *p* = 0.004), suggesting that the magnitude of the indirect effect varied across differing levels of psychological capital. The pattern demonstrated that individuals with higher psychological capital exhibited stronger indirect association, supporting H4’s prediction of moderated mediation.

**Table 4:** Conditional indirect effects of moderated mediation

Psychological Capital Level	β	SE	95% Bias-Corrected CI		*p*
			Lower	Upper	
High (+ 1SD )​	-0.128	0.030	-0.193	-0.073	< 0.001
Low ( -1SD )​	-0.085	0.024	-0.143	-0.045	< 0.001
Index of Moderated Mediation	-0.043	0.020	-0.092	-0.013	0.004

*CI* Confidence Interval

*β =* standardized path coefficient

## Discussion

This research aimed to delve into the connection between relative deprivation and forgiveness, as well as to examine the relational patterns that characterize how and when relative deprivation is associated with forgiveness among college students. As expected, the findings demonstrated a negative association between relative deprivation and forgiveness, in which ego depletion functioned as a mediator while psychological capital operated as a moderator. Moreover, the mediating role of ego depletion varied across different psychological capital degrees.

While exploratory in nature, this study was among the first to reveal that relative deprivation was associated with lower levels of forgiveness in a sample consisting of Chinese university students, complementing the psychological correlates typically examined, such as depression and anxiety [26]. Through establishing this link, our findings contribute to understanding the associations between relative deprivation and psychological adjustment through the lens of forgiveness. Specifically, the results indicated that forgiveness, a key marker of psychological well-being [88], tended to be lower among students reporting higher levels of relative deprivation alongside affective and cognitive disruptions.This negative association between relative deprivation and forgiveness was further reflected in reduced prosocial engagement among students with lower forgiveness.

Our research aligned with broader studies demonstrating that perceived inequality correlates with greater psychological distress [26] and less stable interpersonal relationships [38]. Importantly, our findings add to this body of work through the observed association between relative deprivation - a macro-level socio-environmental variable - and forgiveness among Chinese university students. This study widened the scope of inquiry in showing that factors related to limited forgiveness extended beyond specific interpersonal offenses to macro-level perceptions of social inequality and disadvantage. The integration of socio-environmental elements alongside the traditionally examined individual-level factors enriches our understanding of the contextual factors relevant to forgiveness. Our results revealed that relative deprivation was associated with notable challenges in the process of forgiveness. Consequently, educators could consider addressing not only the interpersonal conflicts that occur alongside specific offenses but also the broader socio-environmental elements that relate to reduced forgiveness, particularly students’ perceived inferior status in social comparisons on campus.

Ego depletion exhibited a mediating role between relative deprivation and forgiveness, contributing to prior research on self-regulation and prosocial behavior. Drawing on the theory of self-regulation as a finite resource [40], our results showed that relative deprivation was associated with higher levels of ego depletion, which was also negatively associated with forgiveness. This empirical pattern suggests that individuals experiencing relative deprivation may expend substantial self-regulatory resources to manage negative cognitions and emotions, and also showed lower capacity for forgiveness - a process that itself involves considerable self-regulation. This pattern of associations reveals that socio-environmental stressors like relative deprivation are associated with interpersonal reconciliation via ego depletion and self-regulatory capacity, underscoring the potentially broad relevance of perceived inequality for interpersonal outcomes. Unlike previous studies that have primarily emphasized mediators like self-esteem [89] and anger [90], this study focused on self-regulatory depletion as a potential statistical mediator echoing prior work on self-control failure [46]. Beyond forgiveness, research further suggests that ego depletion also coincides with higher vulnerability to antisocial responses, including aggression [47]. While our investigation centered on forgiveness, this broader pattern implies that relative deprivation may be pertinent to a range of interpersonal outcomes in contexts of self-regulatory depletion, offering a novel direction for future research in contexts involving social comparison and perceived inequality.

The moderating role of psychological capital demonstrated in our investigation warrants particular attention. Extant research has suggested psychological capital is associated with reduced adverse impacts from stress factors on mental health indicators [91]. However, our findings revealed a nuanced pattern: while individuals with higher psychological capital showed lower overall levels of ego depletion, the strength of the association between relative deprivation and ego depletion was was unexpectedly greater in this group. Notably, this does not negate the protective value of psychological capital. Our results suggested that psychological capital was associated with two distinct patterns: (a) lower baseline levels of depletion, and (b) a stronger association between relative deprivation and ego depletion, possibly reflecting heightened sensitivity to inequity. This dual pattern highlighted the context-dependent associations involving psychological capital - a crucial consideration for understanding its role under different stress conditions.

Several theoretical mechanisms may explain this unexpected pattern. Expectancy violations theory [92, 93] suggests that when outcomes fall short of heightened expectations, individuals experience more severe psychological impact. Those with higher psychological capital may hold stronger expectations for fairness and achievement due to their inherent optimism and self-efficacy, and may be more vulnerable when these expectations are unmet. Similarly, goal-setting theory [94] indicates that individuals with higher standards become more dissatisfied when goals are not achieved, as they invest greater psychological resources in pursuing these elevated aspirations.This pattern suggests that the confidence and optimism of high psychological capital individuals may be associated with intensified psychological distress when reality disappoints, and this distress is also associated with greater self-regulatory effort and ego depletion.

These mechanisms align with related theoretical frameworks that broaden our comprehension of how psychological assets function under adversity. The stress vulnerability hypothesis [95] suggests that the buffering capacity of positive attributes gradually weakens when stress or risk elements exceed critical thresholds. Supporting evidence comes from studies showing that individuals with higher hope experience stronger negative effects from peer victimization [96], and that gratitude’s protective benefits diminish under severe stress [97]. Similarly, protective factors like resilience tend to lose their mitigating influence when risks reach critical levels [98, 99]. Additionally, the reverse stress-buffering model [100] provides a complementary perspective, positing that positive psychological resources may even become counterproductive under extreme adversity [101, 102]. This does not negate the value of psychological capital but highlights its conditional nature. While our cross-sectional design precludes definitive causal inferences, our findings align with these theoretical frameworks. The observed pattern suggested that individuals with higher psychological capital, while generally showing lower overall levels of ego depletion, exhibited heightened vulnerability to the depleting relationship in the context of relative deprivation. The very optimism and confidence that characterize high psychological capital may be linked to greater sensitivity to perceived inequality, aligning with greater ego depletion in this context. Our findings suggested that psychological capital exhibits a dual nature: while it typically serves as a protective resource, it can paradoxically be present alongside greater vulnerability under specific stressful conditions. Such context-dependent patterns highlight the necessity of incorporating situational variables when examining the role of positive psychological attributes.

Building on these theoretical frameworks, this investigation offered empirical evidence regarding the complexities of stress and its interaction with psychological resources. Theoretically, our research expanded understanding of the psychological correlates of relative deprivation by unveiling patterns potentially linking it to forgiveness. By integrating ego depletion as a mediating process, the present study extended self-regulatory frameworks to encompass social comparison phenomena, presenting a differentiated viewpoint concerning.the association between self-regulatory resource depletion and lower prosocial tendencies. Furthermore, our exploration of psychological capital as a moderator highlighted its conditional efficacy, demonstrating that its protective characteristics were less apparent under substantial stress. This finding adds complexity to the assumption of psychological capital’s general benefits, contributing to theoretical discourse on its limitations in high-stress environments.

Practically speaking, this study afforded useful insights into improving student well-being and cultivating a harmonious campus atmosphere. Given the association between relative deprivation and reduced forgiveness observed in this study, administrators may consider fostering fairness and belonging by implementing diverse recognition programs to address issues related to social comparison. Additionally, intervention approaches like cognitive-behavioral therapy (CBT) workshops can assist vulnerable students in reconstructing their self-perceptions and in viewing their environment more positively [103]. Moreover, enhancing self-regulatory resources among students should be prioritized. Interventions like mindfulness training [104], regular physical activity [105], mood regulation strategies [106], and self-affirmation exercises [107] can significantly strengthen self-control. Importantly, these results indicated that the efficacy of psychological capital is context-dependent and may diminish under severe stress [96]. Therefore, administrators should not overstate the benefits of positive psychological attributes. Instead, additional emphasis must be placed on fostering external support mechanisms, such as counselor-led programs and peer support networks, to provide comprehensive social support [91]. Such external support, in combination with enhanced psychological capital, may effectively strengthen students’ resilience and adaptive capacities.

### Limitations

Nevertheless, it is essential to address certain limitations inherent in this study. First, while the validated model is built on strong theoretical foundations and supported by substantial prior research, the cross-sectional design precludes causal inferences, as our findings reflect associations rather than causation among variables. Subsequent research could benefit from employing longitudinal or experimental approaches to verify causal mechanisms in the theoretical model and examine changes in forgiveness over time, along with the roles of ego depletion and psychological capital during this process.

Second, while supplementary validation initially supports the stability characteristics of the ego depletion scale, future research ought to adopt more systematic methods such as test-retest reliability and longitudinal tracking to further verify the temporal stability of these constructs. Future studies may also benefit from developing instruments that better capture trait-like self-regulatory capacity.

Additionally, the association between ego depletion and forgiveness was found to be partial, indicating that other factors, such as anger, self-esteem, and cognitive reappraisal, may play a meaningful role in this dynamic and warrant further investigation. Furthermore, personality traits and social support may be considered as potential moderators, offering promising avenues for capturing individual differences and contextual influences.

Finally, the exclusive recruitment of university students from Shandong Province, China, constrains the external validity of our results. Further investigations would benefit from incorporating more heterogeneous populations spanning different geographical locations and cultural contexts to strengthen the findings’ applicability and reliability.

## Conclusions

This research investigated the association between relative deprivation and forgiveness, revealing that ego depletion served as a statistical mediator while psychological capital played a moderating role. Particularly, higher levels of perceived deprivation were associated with reduced forgiveness. The results revealed that relative deprivation was associated with lower levels of forgiveness via its association with ego depletion, reflecting the link between self-regulatory resource depletion and forgiveness. Additionally, psychological capital moderated the association between relative deprivation and ego depletion, with this moderating effect being more pronounced among college students with elevated psychological capital. Psychological capital also moderated the indirect association from relative deprivation to forgiveness through ego depletion. These results advance understanding of the associations linking perceived inequality to interpersonal reconciliation, particularly highlighting that self-regulatory vulnerability to relative deprivation varies with individual resource levels. These results provide insights for developing interventions targeting forgiveness enhancement among students experiencing heightened relative deprivation.

## Data Availability

The data generated during the current study are available from the corresponding author on reasonable request.

## Abbreviations

RD: Relative Deprivation
ED: Ego Depletion
PsyCap: Psychological Capital
FORG: Forgiveness
FES: Family Economic Status
PRD: The Personal Relative Deprivation scale
PCQ: The Psychological Capital Questionnaire

## References

[1] Yu M, Tian F, Cui Q, Wu H. Prevalence and its associated factors of depressive symptoms among Chinese college students during the COVID-19 pandemic. BMC Psychiatry. 2021;21:1–8. 33514336 10.1186/s12888-021-03066-9 PMC7845579

[2] Leung AN, Wong N, Farver JM. Cyberbullying in Hong Kong Chinese students: life satisfaction, and the moderating role of friendship qualities on cyberbullying victimization and perpetration. Pers Individ Differ. 2018;133:7–12.

[3] Long KN, Worthington EL, VanderWeele TJ, Chen Y. Forgiveness of others and subsequent health and well-being in mid-life: a longitudinal study on female nurses. BMC Psychol. 2020;8:1–11. 33004075 10.1186/s40359-020-00470-w PMC7528379

[4] Quintana-Orts C, Rey L, Worthington EL Jr. The relationship between forgiveness, bullying, and cyberbullying in adolescence: A systematic review. Trauma Violence Abuse. 2021;22(3):588–604. 31434555 10.1177/1524838019869098

[5] Molinero C, Bonete S, Crespí P, Sendra Ramos S, González De Abreu AM. Effectiveness of forgiveness training programs in university contexts: a systematic review and meta-analysis. Cogent Educ. 2024;11(1):2378242.

[6] McCullough ME. Forgiveness as human strength: Theory, measurement, and links to well-being. J Soc Clin Psychol. 2000;19(1):43–55.

[7] Fu H, Watkins D, Hui EK. Personality correlates of the disposition towards inter-personal forgiveness: A Chinese perspective. Int J Psychol. 2004;39(4):305–16.

[8] Furman CR, Luo S, Pond RS. A perfect blame: Conflict-promoting attributions mediate the association between perfectionism and forgiveness in romantic relationships. Pers Individ Differ. 2017;111:178–86.

[9] Schumann K. The psychology of offering an apology: Understanding the barriers to apologizingand how to overcome them. Curr Dir Psychol Sci. 2018;27(2):74–8.

[10] Strelan P, McKee IA, Calic D, Cook L, Shaw L. For whom do we forgive? A functional analysis of forgiveness. Pers Relationships. 2013;20(1):124–39.

[11] Toussaint L, Lee JA, Hyun MH, Shields GS, Slavich GM. Forgiveness, rumination, and depression in the united States and korea: a cross-cultural mediation study. J Clin Psychol. 2023;79(1):143–57. 35700333 10.1002/jclp.23376 PMC11216058

[12] Riek BM. Transgressions, guilt, and forgiveness: a model of seeking forgiveness. J Psychol Theol. 2010;38(4):246–54.

[13] Brudek P, Kaleta K. Marital offence-specific forgiveness as mediator in the relationships between personality traits and marital satisfaction among older couples: perspectives on Lars tornstam’s theory of gerotranscendence. Ageing Soc. 2023;43(1):161–79.

[14] Adams GS, Inesi ME. Impediments to forgiveness: victim and transgressor attributions of intent and guilt. J Pers Soc Psychol. 2016;111(6):866–81. 27537273 10.1037/pspi0000070

[15] Su S, Zhang JC, Xia LX. The relationship between group relative deprivation and aggressive collective action online toward deprivation-related provocateurs within the group: the mediating role of hostile feelings. Curr Psychol. 2022;42(29):25246–56. 10.1007/s12144-022-03530-z PMC9382011 35990206

[16] Smith HJ, Pettigrew TF, Pippin GM, Bialosiewicz S. Relative deprivation: a theoretical and meta-analytic review. Pers Soc Psychol Rev. 2012;16(3):203–32. 22194251 10.1177/1088868311430825

[17] Zhao H, Zhang H. How personal relative deprivation influences moral disengagement: the role of malicious envy and honesty-humility. Scand J Psychol. 2022;63(3):246–55. 34750825 10.1111/sjop.12791

[18] Wang M, Chen M, Chen Z. The effect of relative deprivation on aggressive behavior of college students: a moderated mediation model of belief in a just world and moral disengagement. Bmc Psychol. 2023;11(1):272. 37700345 10.1186/s40359-023-01272-6 PMC10496213

[19] Stouffer SA, Suchman EA, DeVinney LC, Starr SA, Williams RM. The American soldier: adjustment during army life. Princeton,NJ: Princeton University Press; 1949.

[20] Runciman WG. Relative deprivation and social justice: A study of attitudes to social inequality in twentieth-century England. Berkeley,CA: University of California Press; 1966.

[21] Walker I, Smith HJ, editors. Relative deprivation: Specification, development, and integration. Cambridge,UK: Cambridge University Press; 2002.

[22] Kim H, Callan MJ, Gheorghiu AI, Skylark WJ. Social comparison processes in the experience of personal relative deprivation. J Appl Soc Psychol. 2018;48(9):519–32.

[23] Park HJ, Park YB. Negative upward comparison and relative deprivation: sequential mediators between social networking service usage and loneliness. Curr Psychol. 2023;42:1–11.33519148

[24] Jang K, Park N, Song H. Social comparison on facebook: its antecedents and psychological outcomes. Comput Hum Behav. 2016;62:147–54.

[25] Callan MJ, Kim H, Matthews WJ. Age differences in social comparison tendency and personal relative deprivation. Pers Individ Differ. 2015;87:196–9.

[26] Pettigrew TF. Samuel stouffer and relative deprivation. Soc Psychol Quart. 2015;78(1):7–24.

[27] Worthington EL. A stress-and-coping theory of forgivingness and relevant evidence. In: Worthington EL, editor. Forgiveness and reconciliation. London: Routledge; 2006. pp. 15–83.

[28] Bobocel DR. Coping with unfair events constructively or destructively: the effects of overall justice and self-other orientation. J Appl Psychol. 2013;98(5):720–31. 23668596 10.1037/a0032857

[29] Barclay LJ, Saldanha MF. Facilitating forgiveness in organizational contexts: exploring the injustice gap, emotions, and expressive writing interventions. J Bus Ethics. 2016;137:699–720.

[30] Strelan P, Fiore CD, Prooijen JW. The empowering effect of punishment on forgiveness. Eur J Soc Psychol. 2017;47:472–87.

[31] Riek BM, Mania EW. The antecedents and consequences of interpersonal forgiveness: a meta-analytic review. Pers Relationships. 2012;19(2):304–25.

[32] Karremans JC, Regalia C, Paleari FG, Fincham FD, Cui M, Takada N, et al. Maintaining harmony across the globe: the cross-cultural association between closeness and interpersonal forgiveness. Soc Psychol Pers Sci. 2011;2(5):443–51.

[33] Merolla AJ, Zhang S, Sun S. Forgiveness in the united States and china: antecedents, consequences, and communication style comparisons. Commun Res. 2013;40:595–622.

[34] Ho MY, Fung HH. A dynamic process model of forgiveness: a cross-cultural perspective. Rev Gen Psychol. 2011;15(1):77–84.

[35] Ho MY. Forgiving in East Asian cultures: theory and empirical research. In: Worthington EL, editor. Handbook of forgiveness. London: Routledge; 2019. pp. 234–41.

[36] Guimond S, Dambrun M. When prosperity breeds intergroup hostility: the effects of relative deprivation and relative gratification on prejudice. Pers Soc Psychol Bull. 2002;28(7):900–12.

[37] Smith HJ, Huo YJ. Relative deprivation: how subjective experiences of inequality influence social behavior and health. Policy Insights Behav Brain Sci. 2014;1(1):231–8.

[38] Lin X, Wu CH, Dong Y, Chen GZ, Wei W, Duan J. Psychological contract breach and destructive voice: the mediating effect of relative deprivation and the moderating effect of leader emotional support. J Vocat Behav. 2022;135:103720.

[39] DeWall CN, Baumeister RF, Gailliot MT, Maner JK. Depletion makes the heart grow less helpful: helping as a function of self-regulatory energy and genetic relatedness. Pers Soc Psychol Bull. 2008;34(12):1653–62. 19050337 10.1177/0146167208323981

[40] Baumeister RF, Bratslavsky E, Muraven M, Tice DM. Ego depletion: is the active self a limited resource? J Pers Soc Psychol. 1998;74(5):1252–65. 9599441 10.1037//0022-3514.74.5.1252

[41] Muraven M, Baumeister RF. Self-regulation and depletion of limited resources: does self-control resemble a muscle? Psychol Bull. 2000;126(2):247–59. 10748642 10.1037/0033-2909.126.2.247

[42] Baumeister RF. Self-regulation, ego depletion, and Inhibition. Neuropsychologia. 2014;65:313–9. 25149821 10.1016/j.neuropsychologia.2014.08.012

[43] Osborne D, Sibley CG, Sengupta NK. Income and neighborhood-level inequality predict self-esteem and ethnic identity centrality through individual- and group-based relative deprivation: a multilevel path analysis. Eur J Soc Psychol. 2015;45(3):368–77.

[44] Inzlicht M, Kang SK. Stereotype threat spillover: how coping with threats to social identity affects aggression, eating, decision making, and attention. J Pers Soc Psychol. 2010;99(3):467–81. 20649368 10.1037/a0018951

[45] Smith HJ, Ryan D, Jaurique A, Duffau E. Personal relative deprivation and mental health among university students: Cross-sectional and longitudinal evidence. Anal Soc Iss Pub Pol. 2020;20(1):287–314.

[46] Baumeister RF, Vohs KD. Strength model of self-regulation as limited resource: Assessment, controversies, update. In: Vohs KD, Baumeister RF, editors. Self-regulation and self-control. London: Routledge; 2018. pp. 78–128.

[47] DeWall CN, Baumeister RF, Stillman TF, Gailliot MT. Violence restrained: effects of self-regulation and its depletion on aggression. J Exp Soc Psychol. 2007;43(1):62–76.

[48] Muraven M. Prejudice as self-control failure. J Appl Soc Psychol. 2008;38(2):314–33.

[49] Yam KC, Fehr R, Keng-Highberger FT, Klotz AC, Reynolds SJ. Out of control: A self-control perspective on the link between surface acting and abusive supervision. J Appl Soc Psychol. 2016;101(2):292–301. 10.1037/apl0000043 26214087

[50] Trougakos JP, Beal DJ, Cheng BH, Hideg I, Zweig D. Too drained to help: a resource depletion perspective on daily interpersonal citizenship behaviors. J Appl Soc Psychol. 2015;100(1):227–36. 10.1037/a0038082 25314365

[51] Jin HS, Kim HJ, Suh J, Sheehan B, Meeds R. Ego depletion and charitable support: the moderating role of self-benefit and other-benefit charitable appeals. J Advert. 2021;50(4):479–93.

[52] Pohl RF, Erdfelder E, Hilbig BE, Liebke L, Stahlberg D. Effort reduction after self-control depletion: the role of cognitive resources in use of simple heuristics. J Cogn Psychol. 2013;25(3):267–76.

[53] Payne JW, Bettman JR, Johnson EJ. Adaptive strategy selection in decision making. J Exp Psychol Learn. 1988;14(3):534–52.

[54] Lazarus RS, Folkman S. Stress, appraisal, and coping. New York: Springer; 1984.

[55] Luthans F, Youssef CM, Avolio BJ. Psychological capital: developing the human competitive edge. New York: Oxford University Press; 2007.

[56] Newman A, Ucbasaran D, Zhu FEI, Hirst G. Psychological capital: A review and synthesis. J Organ Behav. 2014;35(S1):S120–38.

[57] Hagger MS, Wood C, Stiff C, Chatzisarantis NL. The strength model of self-regulation failure and health-related behaviour. Health Psychol Rev. 2009;3(2):208–38.

[58] Hagger MS, Wood C, Stiff C, Chatzisarantis NL. Ego depletion and the strength model of self-control: a meta-analysis. Psychol Bull. 2010;136(4):495–525. 20565167 10.1037/a0019486

[59] Hobfoll SE. Social and psychological resources and adaptation. Rev Gen Psychol. 2002;6(4):307–24.

[60] Hobfoll SE, Halbesleben J, Neveu JP, Westman M. Conservation of resources in the organizational context: the reality of resources and their consequences. Annu Rev Organ Psychol Organ Behav. 2018;5:103–28.

[61] Chen S, Westman M, Hobfoll SE. The commerce and crossover of resources: resource conservation in the service of resilience. Stress Health. 2015;31(2):95–105. 25873421 10.1002/smi.2574 PMC4564014

[62] Roberts SJ, Scherer LL, Bowyer CJ. Job stress and incivility: what role does psychological capital play? J Leadersh Org Stud. 2011;18(4):449–58.

[63] Brummelhuis LL, Ter Hoeven CL, Bakker AB, Peper B. Breaking through the loss cycle of burnout: the role of motivation. J Occup Organ Psychol. 2011;84(2):268–87.

[64] Cheung F, Tang CS, Tang S. Psychological capital as a moderator between emotional labor, burnout, and job satisfaction among school teachers in China. Int J Stress Manag. 2011;18(4):348–71.

[65] Kuvaas B, Buch R, Weibel A, Dysvik A, Nerstad CG. Do intrinsic and extrinsic motivation relate differently to employee outcomes? J Econ Psychol. 2017;61:244–58.

[66] Darvishmotevali M, Ali F. Job insecurity, subjective well-being and job performance: the moderating role of psychological capital. Int J Hosp Manag. 2020;87:102462.

[67] Curran PG. Methods for the detection of carelessly invalid responses in survey data. J Exp Soc Psychol. 2016;66:4–19.

[68] Fleeson W, Jayawickreme E. Whole trait theory. J Res Pers. 2015;56:82–92. 26097268 10.1016/j.jrp.2014.10.009 PMC4472377

[69] Mischel W, Shoda Y. A cognitive-affective system theory of personality: reconceptualizing situations, dispositions, dynamics, and invariance in personality structure. Psychol Rev. 1995;102(2):246–68. 7740090 10.1037/0033-295x.102.2.246

[70] Luthans F, Norman SM, Avolio BJ, Avey JB. The mediating role of psychological capital in the supportive organizational climate - employee performance relationship. J Organiz Behav. 2008;29(2):219–38.

[71] Baumeister RF. Willpower:Rediscovering the greatest human strength (2nd ed). New York: Penguin Books; 2024.

[72] Schmeichel BJ, Zell A. Trait self-control predicts performance on behavioral tests of self-control. J Pers. 2007;75(4):743–56. 17576357 10.1111/j.1467-6494.2007.00455.x

[73] Ma A. Relative deprivation and social adaptation: the role of mediator and moderator. Acta Psychol Sin. 2012;44(3):377–87.

[74] Lin SH, Johnson RE. A suggestion to improve a day keeps your depletion away: examining promotive and prohibitive voice behaviors within a regulatory focus and ego depletion framework. J Appl Psychol. 2015;100(5):1381–97. 25706447 10.1037/apl0000018

[75] Kuo Z, Sai Z, Yinghong D. Positive psychological capital: measurement and relationship with mental health. Stud Psychol Behav. 2010;8(1):58–64.

[76] Yang X, Li W, Zheng X. Development of trait forgiveness questionnaire for college students. Psychol Tech Appl. 2019;7(8):472–84.

[77] Fehr R, Gelfand MJ, Nag M. The road to forgiveness: a meta-analytic synthesis of its situational and dispositional correlates. Psychol Bull. 2010;136(5):894–914. 20804242 10.1037/a0019993

[78] Da Silva S, Matsushita R, Ludwig R, Bellincanta L. Ego depletion May explain gender differences in multitasking. J Interdiscip Econ. 2021;33(1):130–9.

[79] Anderson JC, Gerbing DW. Structural equation modeling in practice: A review and recommended two-step approach. Psychol Bull. 1988;103(3):411–23.

[80] Little TD, Rhemtulla M, Gibson K, Schoemann AM. Why the items versus parcels controversy needn’t be one. Psychol Methods. 2013;18(3):285–300. 23834418 10.1037/a0033266 PMC3909043

[81] Sinacore J. Multiple regression: testing and interpreting interactions. Eval Pract. 1993;14:167–8.

[82] Podsakoff PM, MacKenzie SB, Lee JY, Podsakoff NP. Common method biases in behavioral research: a critical review of the literature and recommended remedies. J Appl Psychol. 2003;88(5):879–903. 14516251 10.1037/0021-9010.88.5.879

[83] Kock N. Common method bias in PLS-SEM: A full collinearity assessment approach. Int J e-Collaboration. 2015;11(4):1–10.

[84] Wheaton B, Muthen B, Alwin DF, Summers GF. Assessing reliability and stability in panel models. Sociol Methodol. 1977;8:84–136.

[85] Hu L, Bentler PM. Cutoff criteria for fit indexes in covariance structure analysis: conventional criteria versus new alternatives. Struct Equ Model. 1999;6(1):1–55.

[86] Browne MW, Cudeck R. Alternative ways of assessing model fit. Sociol Method Res. 1992;21(2):230–58.

[87] Shevlin M, Miles JNV. Effects of sample size, model specification and factor loadings on the GFI in confirmatory factor analysis. Pers Indiv Differ. 1998;25(1):85–90.

[88] Worthington EL, Scherer M. Forgiveness is an emotion-focused coping strategy that can reduce health risks and promote health resilience: Theory, review, and hypotheses. Psychol Health. 2004;19(3):385–405.

[89] Peng J, Zhang J, Xia Z, Wang X, Dan Z, Zheng S, Lv J. How does relative deprivation relate to aggression in young male migrant workers? The mediator of self-esteem. Curr Psychol. 2023; 42:8136-8143 .

[90] Cen YS, Xia LX. Serial cascade effects of relative deprivation and anger rumination on the development of social aggression over 2.5 years in emerging adults. J Youth Adolescence. 2024; 53 :2762-2775. 10.1007/s10964-024-02029-z 38849686

[91] Wang H, Ng TK, Siu OL. How does psychological capital lead to better well-being for students? The roles of family support and problem-focused coping. Curr Psychol. 2023;42(26):22392–403. 10.1007/s12144-022-03339-w PMC9209831 35756898

[92] Burgoon JK, Jones SB. Toward a theory of personal space expectations and their violations. Hum Commun Res. 1976;2(2):131–46.

[93] Burgoon JK. Interpersonal expectations, expectancy violations, and emotional communication. J Lang Soc Psychol. 1993;12(1–2):30–48.

[94] Locke EA, Latham GP. A theory of goal setting and task performance. Englewood Cliffs,NJ: Prentice Hall; 1990.

[95] Scharf SH, Schmidt MV. Animal models of stress vulnerability and resilience in translational research. Curr Psychiat Rep. 2012;14:159–65. 10.1007/s11920-012-0256-0 22278810

[96] Xiang B, Wei C, Han X, Zheng H, Liu J. .Relationship between peer victimization and children’s depression: the moderating effects of individual and Group-level hope. Chin J Clin Psychol. 2024;32(1):76–81.

[97] Li D, Zhang W, Li X, Li N, Ye B. Gratitude and suicidal ideation and suicide attempts among Chinese adolescents: Direct, mediated, and moderated effects. J Adolescence. 2012;35(1):55–66. 10.1016/j.adolescence.2011.06.005 21774977

[98] Gutman LM, Sameroff AJ, Cole R. Academic growth curve trajectories from 1st grade to 12th grade: effects of multiple social risk factors and preschool child factors. Dev Psychol. 2003;39(4):777. 12859129 10.1037/0012-1649.39.4.777

[99] Vanderbilt-Adriance E, Shaw DS. Conceptualizing and re-evaluating resilience across levels of risk, time, and domains of competence. Clin Child Fam Psych. 2008;11:30–58. 10.1007/s10567-008-0031-2 PMC2683037 18379875

[100] Rueger SY, Malecki CK, Pyun Y, Aycock C, Coyle S. A meta-analytic review of the association between perceived social support and depression in childhood and adolescence. Psychol Bull. 2016;142(10):1017. 27504934 10.1037/bul0000058

[101] Liu C, Huang N, Ahmed F, Shahid M, Wang X, Guo J. The reverse buffering effects of social support on the relationships between stresses and mental health: a survey of Chinese adults during the COVID-19 lockdown. Eur J Psychotraumatol. 2021;12(1):1952777. 34408816 10.1080/20008198.2021.1952777 PMC8366626

[102] Li Y, Li D, Li X, Zhou Y, Sun W, Wang Y, Li J. Cyber victimization and adolescent depression: the mediating role of psychological insecurity and the moderating role of perceived social support. Child Youth Serv Rev. 2018;94:10–9.

[103] Creed A, Waltman TH, A Frankel S, Williston SA. School-based cognitive behavioral therapy: current status and alternative approaches. Curr Psychiat Res Re. 2016;12(1):53–64.

[104] Brockman R, Ciarrochi J, Parker P, Kashdan T. Emotion regulation strategies in daily life: Mindfulness, cognitive reappraisal and emotion suppression. Cogn Behav Therapy. 2017;46(2):91–113. 10.1080/16506073.2016.1218926 27684649

[105] Best JR, Nagamatsu LS, Liu-Ambrose T. Improvements to executive function during exercise training predict maintenance of physical activity over the following year. Front Hum Neurosci. 2014;8:353. 24904387 10.3389/fnhum.2014.00353 PMC4034407

[106] Scarantino A. Exploring the roles of emotions in self-Control. Oxford,UK:Oxford University Press; 2020.

[107] Cascio CN, O’Donnell MB, Tinney FJ, Lieberman MD, Taylor SE, Strecher VJ, Falk EB. Self-affirmation activates brain systems associated with self-related processing and reward and is reinforced by future orientation. Soc Cogn Affect Neur. 2016;11(4):621–9. 10.1093/scan/nsv136 PMC4814782 26541373

